# Improving Psychiatric History-Taking Documentation: A Quality Improvement Audit and Re-Audit

**DOI:** 10.7759/cureus.108114

**Published:** 2026-05-01

**Authors:** Qaisar Farooq, Tahir Saleem

**Affiliations:** 1 Psychiatry, St. Lukes Hospital, Kilkenny, IRL

**Keywords:** clinical audit, documentation standards, mental health services, patient safety, psychiatric history taking, quality improvement

## Abstract

Purpose: Accurate psychiatric history taking is fundamental to diagnosis, formulation, treatment planning, and patient safety. This comparative audit evaluated the impact of training interventions on the completeness of psychiatric history documentation, compared outcomes across two audit cycles, and identified ongoing documentation deficits.

Patients and methods: A clinical audit and re-audit were carried out in a community mental health service. The first audit cycle took place in July 2025 and the second in December 2025. In each cycle, 10 first-assessment psychiatric records of newly referred patients were reviewed. Each record was assessed according to the psychiatric history-taking domains described in Kaplan and Sadock’s *Comprehensive Textbook of Psychiatry.*

Results: The re-audit showed improvements in several documentation domains. Personal history and social history increased from 30% and 50% in cycle 1 to 100% in cycle 2, while past psychiatric history improved from 80% to 100%. Presenting complaint and history of presenting illness remained stable at 100% and 90%, respectively. Mental state examination increased from 70% to 90%, and legal/forensic history improved from 40% to 50%. Identifying information and risk assessment remained unchanged at 80% and 80%, respectively. Some areas showed little improvement, with formulation increasing only slightly from 0% to 10% and alcohol history rising from 20% to 30%. Documentation in essential areas declined, with current medications falling from 30% to 10% and collateral history dropping from 70% to 20%.

Conclusion: Training helped improve the documentation of psychiatric history, but some areas remained incomplete. Ongoing supervision, experiential learning, and the use of structured templates are essential to sustaining improvements and ensuring that documentation remains comprehensive and accurate.

## Introduction

Background

Accurate and comprehensive psychiatric history taking is essential for diagnosis, formulation, treatment planning, and effective clinical management [1-3]. Well-documented histories support high-quality patient care, ensure medico-legal accountability, and meet professional standards set by regulatory bodies [4]. Omissions in key areas may result in gaps in care or reduce the overall quality of patient management. A preliminary assessment within the local service highlighted several areas where psychiatric history documentation was incomplete. To address this, the Kilkenny East Community Mental Health Team planned an audit to evaluate the quality and completeness of psychiatric history records, identify areas of incomplete documentation, explore possible reasons for these gaps, and update the existing history-taking template. Previous quality improvement studies have shown that structured templates and audit-feedback cycles can improve documentation completeness; however, variability persists across domains, particularly in safety-critical areas such as medication and collateral history documentation [5-7]. There is limited local data on documentation quality within community mental health services in Ireland. This audit was undertaken to assess baseline documentation, implement a targeted intervention, and evaluate changes across audit cycles within a real-world clinical setting.

Objectives

This study aimed to assess the completeness of psychiatric history documentation within a community mental health service following a teaching and feedback intervention. It compared documentation patterns between two audit cycles (July 2025 and December 2025) to identify changes across domains, with particular attention to safety-critical areas such as medication and collateral history. This was conducted as a pilot quality improvement audit to explore documentation practices and identify areas for further improvement.

## Materials and methods

The audit was conducted in the Kilkenny East Community Mental Health Team, a community-based adult psychiatric service. First-assessment records of newly referred adult patients were reviewed. Documentation was completed by doctors within the team responsible for initial psychiatric assessments, and the same group of clinicians contributed to both audit cycles.

The first audit cycle was conducted in July 2025 and the second in December 2025. The interval allowed implementation of a teaching and feedback intervention. Following Cycle 1, the results were presented to the clinical team during a structured session, highlighting incomplete domains and emphasizing key areas such as medication history, collateral history, risk assessment, and formulation. The existing history-taking template was also reviewed. The intervention consisted of a single teaching session with informal reinforcement in routine clinical practice.

Documentation was assessed using a structured audit proforma derived from domains described in standard psychiatric textbooks [1,2]. The tool was adapted into a checklist format for local use and was not formally validated. Each domain was scored as either “complete” or “incomplete.” A domain was considered complete only if all predefined subheadings were documented; if any required element was missing or insufficiently recorded, the domain was classified as incomplete. This approach was applied consistently across all records to minimize subjectivity. The audit assessed completeness of documentation rather than qualitative depth. The proportion of completed domains was calculated and compared descriptively between the two audit cycles.

Psychiatric history-taking audit proforma

The audit used a structured psychiatric history-taking proforma covering all key domains for initial assessment documentation. Each domain included multiple subheadings, as shown in Table 1.

**Table 1:** Audit proforma Each domain of the psychiatric history-taking proforma was evaluated and scored as either complete or incomplete. A domain was considered complete if all its subheadings were fully documented according to the selected standards. If any subheading was missing, incomplete, or insufficiently documented, the domain was scored as incomplete. OTC: Over-the-counter; MCRN: Medical council registration number.

Domain	Examples of Subheadings	Scoring
Identifying information	Sticker, date/time, source of referral, GP details	Complete/Incomplete
Presenting complaint	Patient’s own words, ideally quoted	Complete/Incomplete
History of present Illness	Chronology, onset, duration, severity, functional impact, and explanatory model	Complete/Incomplete
Past psychiatric history	Diagnoses, hospitalizations, treatments, outcomes, and self-harm/violence	Complete/Incomplete
Medical/surgical history	Major illnesses, surgeries, and head injuries	Complete/Incomplete
Allergies	Drug/food allergies	Complete/Incomplete
Current medications	All prescribed and OTC meds, doses, adherence, and side effects	Complete/Incomplete
Substance use history	Alcohol, tobacco, illicit/prescription drugs, and consequences	Complete/Incomplete
Family history	Psychiatric disorders, suicide, and medical illnesses	Complete/Incomplete
Personal history	Birth, schooling, relationships, trauma, and marriage/children	Complete/Incomplete
Social history	Living situation, employment, financial, and legal	Complete/Incomplete
Mental status exam	Appearance, behavior, speech, mood, cognition, and insight	Complete/Incomplete
Risk assessment	Suicidal/homicidal ideation, self-neglect, and risk to others	Complete/Incomplete
Diagnosis	Primary diagnosis and differential/comorbid conditions	Complete/Incomplete
Formulation	Predisposing, precipitating, perpetuating, and protective factors	Complete/Incomplete
Treatment plan	Immediate and long-term plan	Complete/Incomplete
Attending doctor details	Name, signature, MCRN, and date/time	Complete/Incomplete

## Results

The re-audit showed significant gains in several core areas of psychiatric history documentation while also revealing persistent and new areas of incomplete recording.

Documentation of personal history improved from 3/10 (30%) in Cycle 1 to 10/10 (100%) in Cycle 2. Social history increased from 5/10 (50%) to 10/10 (100%). Past psychiatric history improved from 8/10 (80%) to 10/10 (100%), and the presenting complaint increased from 9/10 (90%) to 10/10 (100%). Identifying information, including patient identification stickers, GP details, referral source, and date/time of assessment, remained unchanged at 8/10 (82.5%) in both cycles.

Some areas showed limited improvement. Risk assessment remained stable at 8/10 (80%). Formulation improved marginally from 0/10 (0%) to 1/10 (10%). Alcohol history increased slightly from 2/10 (20%) to 3/10 (30%). These gaps may reflect ongoing pressures such as time constraints and clinician workload.

However, documentation in certain domains declined. Current medications decreased from 3/10 (30%) to 1/10 (10%), and collateral history fell from 7/10 (70%) to 2/10 (20%), identifying new areas requiring targeted intervention. The comparison of documentation compliance between the two audit cycles is presented in Table 2.

**Table 2:** Comparison of documentation compliance between audit cycles

Documentation Domain	Cycle 1 (July 2025), n (%)	Cycle 2 (December 2025), n (%)
Identifying information (combined)	8 (80%)	8 (80%)
Presenting complaint	9 (90%)	10 (100%)
History of present Illness	9 (90%)	9 (90%)
Mental state examination	7 (70%)	9 (90%)
Past psychiatric history	8 (80%)	10 (100%)
Personal history	3 (30%)	10 (100%)
Social history	5 (50%)	10 (100%)
Current medications	3 (30%)	1 (10%)
Alcohol history	2 (20%)	3 (30%)
Collateral history	7 (70%)	2 (20%)
Risk assessment	8 (80%)	8 (80%)
Legal/forensic history	4 (40%)	5 (50%)
Formulation	0 (0%)	1 (10%)

## Discussion

This audit identified changes in documentation patterns following a structured training and feedback intervention. Improvements were observed in several domains, including personal history, social history, and past psychiatric history. Previous studies have shown that structured documentation frameworks and targeted educational interventions can improve the completeness and reliability of clinical records and enhance patient safety [5-7]. These findings suggest that structured approaches may support improvement in domains that are more descriptive and closely aligned with template-based documentation.

However, improvement was not consistent across all domains. Documentation declined in key safety-related areas, particularly current medications (3/10 to 1/10) and collateral history (7/10 to 2/10). These findings are clinically significant and represent an important limitation of the intervention. Medication documentation is a fundamental safety requirement rather than a complex task requiring higher-order reasoning, and its decline indicates a gap in clinical prioritization rather than difficulty in synthesis. Similarly, the reduction in collateral history suggests a loss of clinically relevant information that is essential for accurate psychiatric assessment.

Given the small sample size (n = 10 per cycle), small numerical differences between cycles (e.g., 0/10 to 1/10 or 2/10 to 3/10) should be interpreted cautiously, as they may not represent meaningful changes in documentation practice. These variations may reflect individual case differences rather than true improvements.

One possible explanation is that the intervention may have inadvertently emphasized completion of structured or narrative sections, such as personal and social history, without adequately reinforcing safety-critical domains. This may have led to a shift in focus toward template completion rather than clinical prioritization. While previous studies suggest that structured documentation systems can improve completeness [8,9], they may also risk promoting a checklist-driven approach if not balanced with clinical emphasis on patient safety.

Strengthening experiential learning through repeated audit cycles, structured supervision, and targeted teaching sessions may help address these gaps. Educational strategies such as simulation-based training and structured clinical assessments have been shown to improve clinical reasoning and documentation skills among trainees [10]. Additionally, quality improvement research has demonstrated that regular audit and feedback mechanisms can modify professional behavior and improve compliance with documentation standards [11]. Structured documentation templates and electronic prompts may support clinicians in translating clinical reasoning into written records; however, these tools must explicitly prioritize safety-critical domains to avoid unintended neglect of essential clinical information [12].

Workload pressures and documentation burden remain important contextual factors influencing record completeness. Studies examining electronic health record use have shown that increasing administrative workload can negatively affect documentation quality [13,14]. However, the observed improvement in more time-intensive domains such as personal and social history suggests that time constraints alone do not fully explain the decline in medication and collateral documentation. This indicates that prioritization within the intervention may have played a more significant role than workload alone.

Overall, these findings suggest that while structured training may support improvement in some documentation domains, it may also have unintended effects if safety-critical areas are not explicitly emphasized. Future quality improvement efforts should focus on targeted reinforcement of medication documentation and collateral history, clearer prioritization within templates, and repeated audit cycles to ensure balanced and clinically meaningful improvement.

Limitations

This study has several limitations. It was a single-site audit with a small sample size (n = 10 per cycle), and findings are descriptive rather than statistically inferential. No statistical analysis was performed. The small sample size increases the impact of minor variations, meaning that changes of 10%-20% may reflect single-case differences rather than true improvements. The binary scoring system may oversimplify documentation quality and does not capture partial completeness or clinical depth. The audit tool, although based on standard psychiatric domains, was not formally validated. While the same clinicians contributed to both cycles, individual variation in documentation practices may still have influenced results. The intervention was limited to a single teaching session and may not have been sufficient to produce sustained changes across all domains. These factors limit generalizability, and findings should be interpreted as a local quality improvement observation.

## Conclusions

This audit identified descriptive changes in psychiatric history documentation following a structured teaching and feedback intervention within a single service. Improvements were observed in several domains, including personal history, social history, and past psychiatric history, reflecting better adherence to standard documentation frameworks. These findings are consistent with existing quality improvement literature suggesting that audit and feedback can support improvements in clinical record-keeping practices. However, improvement was not uniform across all domains. The decline in documentation of current medications and collateral history represents an important safety concern and highlights limitations of the intervention. These findings suggest that structured teaching alone may not be sufficient to ensure comprehensive documentation, particularly in safety-critical areas that require consistent prioritization.

Overall, the results emphasize the need for targeted reinforcement of key clinical domains, alongside structured templates, supervision, and repeated audit cycles. Workload pressures and documentation burden may also influence documentation quality. Given the small sample size and single-site design, these findings should be interpreted as a local quality improvement observation. Further studies with larger samples and more robust methodology are required to evaluate the effectiveness of interventions aimed at improving psychiatric documentation.
